# Chagas Disease Surveillance Activities — Seven States, 2017

**DOI:** 10.15585/mmwr.mm6726a2

**Published:** 2018-07-06

**Authors:** Carolyne Bennett, Anne Straily, Dirk Haselow, Susan Weinstein, Richard Taffner, Hayley Yaglom, Kenneth Komatsu, Heather Venkat, Catherine Brown, Paul Byers, John Dunn, Abelardo Moncayo, Bonny C. Mayes, Susan P. Montgomery

**Affiliations:** ^1^Division of Parasitic Diseases and Malaria, Center for Global Health, CDC; ^2^Arkansas Department of Health; ^3^Arizona Department of Health Services; ^4^Career Epidemiology Field Officer Program, CDC; ^5^Massachusetts Department of Public Health; ^6^Mississippi State Department of Health; ^7^Tennessee Department of Health; ^8^Texas Department of State Health Services.

Chagas disease, a potentially life-threatening disease caused by the protozoan parasite *Trypanosoma cruzi*, has become a concern in the United States as a result of human emigration from Latin America where Chagas disease is endemic (*1*). It is estimated that as many as 8 million people living in Mexico, and Central and South America have Chagas disease.* Most cases of Chagas disease in the United States are chronic infections; however, rare cases of acute congenital infections and autochthonous vectorborne transmission have been reported (*2*). To understand how data are collected and used, a review of state-level public health surveillance for Chagas disease was conducted through semistructured interviews with health officials in six states (Arizona, Arkansas, Louisiana, Mississippi Tennessee, and Texas) where Chagas disease is reportable and one (Massachusetts) where it was previously reportable. States implemented surveillance in response to blood donor screening for Chagas disease and to identify the route of disease transmission. Many states reported primarily chronic cases and had limited ability to respond to local transmission because acute cases were infrequently reported. Surveillance remains important in states with large populations of immigrants or frequent travelers from countries with endemic disease and for states with a risk for local transmission. Surveillance efforts can also help increase awareness among providers and assist in linking patients with Chagas disease to treatment to help prevent cardiac and gastrointestinal complications.

Chagas disease is spread via contact with infected vector insects (triatomines, also known as “kissing bugs”), congenitally, and rarely through organ transplantation or blood transfusion from an infected donor (*3*). *T. cruzi* vectors and infected mammalian reservoirs are found throughout the United States (*2*). The acute stage of Chagas disease is often asymptomatic, or flu-like symptoms will develop that can last up to 2 months after the 1–2-week incubation period (*2*). Infants are at higher risk for developing severe manifestations, such as myocarditis or meningoencephalitis during the acute stage. If untreated, infection becomes chronic. Most patients with chronic infection remain asymptomatic; however, 20%–30% develop cardiac or gastrointestinal complications, which can be fatal (*2*). Chagas disease is likely having an underrecognized impact on the health care system and economy because of limited screening and treatment and a lack of awareness among health care professionals (*4*,*5*). With an undefined prevalence of disease and risk for transmission in the United States, surveillance for Chagas disease could help improve understanding of Chagas disease–associated cardiac morbidity and mortality, gastrointestinal disease, and risk for congenital and autochthonous infections (*6*). Timely recognition and treatment can prevent chronic infection and reduce health care needs.

States where Chagas disease is or was previously listed as a reportable condition were identified using the Council of State and Territorial Epidemiologists database (https://www.cste.org/group/SRCAQueryRes) and state health department websites. After reviewing the surveillance guidelines for each state, a qualitative questionnaire was formulated. Key informant, semistructured interviews were conducted by telephone with epidemiologists from each state to identify why Chagas disease was designated a reportable condition, how cases are reported and by whom, what actions follow identification of a case, and how collected data are used and disseminated. State respondents were also asked whether data were collected on pregnant women at risk, infants born to infected mothers, nonhuman cases, or triatomine vectors.

As of December 2017, Arizona, Arkansas, Louisiana, Mississippi, Tennessee, and Texas conduct surveillance for Chagas disease; Massachusetts discontinued surveillance in 2014. Surveillance activities were primarily stimulated by blood donor screening and are conducted with the purpose of identifying the source of transmission (Table 1). Five of the six states where Chagas disease is reportable are notified of possible cases by blood donor centers, physicians, and laboratories; the majority of reports in most of these states are received from blood donor centers. All states investigate reported cases to determine where the exposure most likely occurred. The primary focus of case investigations in Arizona, Louisiana, Mississippi, and Texas is identification of local autochthonous transmission, whereas Arkansas and Tennessee collect data on all modes of transmission. Four states conduct routine environmental assessments at the patient’s residence if autochthonous exposure is suspected.

**TABLE 1:** Summary of state surveillance for Chagas disease, including year each state began reporting and primary and secondary reasons for initiating surveillance — Chagas disease surveillance activities, seven states,* 2017

State	Year reporting began	Primary objectives for Chagas disease surveillance	Reasons for initiating Chagas disease surveillance
Arizona	2008	Identify source of infection; monitor acute and chronic disease burden	Presence of *T. cruzi*-positive triatomines in the state
Arkansas	2013	Identify source of infection; monitor acute and chronic disease burden	Understand the potential burden of locally acquired, congenital, and imported cases; create awareness among physicians working with populations at risk
Louisiana	2013	Identify source of infection; monitor incident cases	Monitor incident cases; assess risk factors for local autochthonous transmission
Mississippi	2010	Identify source of infection; monitor acute and chronic disease burden	Determine whether cases identified by blood banks are caused by local autochthonous transmission; monitor extent of Chagas disease testing occurring at laboratories throughout the state
Tennessee	2010	Identify source of infection; monitor acute and chronic disease burden	Identification of *T. cruzi*-infected triatomines and nonhuman hosts during a serosurvey
Texas	2013	Identify source of infection; monitor acute and chronic disease burden	Monitor incident cases; assess risk factors for local autochthonous transmission; increase awareness of physicians working with populations at risk
Massachusetts	2008	Monitor chronic disease phase burden	Ensure that blood donors identified through screening are referred for appropriate care

*Information about Massachusetts surveillance of Chagas disease conducted from 2008 to 2014.

The states, with input from CDC, provide education and guidance to physicians regarding the clinical management of Chagas disease. In Arkansas, the health department disseminates Chagas disease health alerts to physicians, particularly obstetricians/gynecologists who care for pregnant women at risk. However, no state conducts surveillance specifically for congenital infections. Five states disseminate surveillance data through a report distributed to health care providers, and all six states post case counts on the state health department website or as an annual disease summary (Table 2).

**TABLE 2:** Methods used to disseminate Chagas disease surveillance data in states where Chagas disease is reportable — six states, 2017

Dissemination methods	Arkansas	Arizona	Louisiana	Mississippi	Tennessee	Texas
Peer-reviewed literature					X	
Report to health care providers	X	X	X	X	X	
Public report/website	X	X	X	X	X	X
In-house report					X	
Other						X*

*Texas Chagas taskforce creates awareness within Texas with subgroups of physicians, veterinarians, and entomologists.

None of the states includes nonhuman data as part of systematic public health surveillance. When Chagas disease surveillance began in Texas in 2013, reports of canine infections were collected for 3 years, but state health officials discontinued this practice after determining that canine infection status was not useful for informing human risk. Although not systematically tracked, most states analyze submitted insects and, depending on classification and likelihood of human contact, send triatomines to CDC for *T. cruzi* testing.

Three states (Arizona, Texas, and Massachusetts) have made changes to their Chagas disease surveillance system since inception. In Arizona, a new case definition was applied in 2016 to classify blood donor cases with respect to confirmatory testing results from the reference diagnostic laboratory at CDC. Texas updated the case definition to collect data on progression from asymptomatic chronic infection to clinical disease in reported cases to better understand the burden of disease on the health care system. In Massachusetts, Chagas disease was added to their reportable condition list in 2008 after the Food and Drug Administration approved the first screening test for *T. cruzi* infection in blood donors and donor screening was initiated. Recognizing that infected donors might be identified through screening and require evaluation and follow-up, Massachusetts public health officials wanted to increase awareness among health care providers in the state to ensure effective referral to care. However, Chagas disease surveillance demonstrated that donors at risk were infrequently identified, and the need for public health response was limited; thus, in 2014, Chagas disease was subsequently removed from the state’s list of reportable conditions.

## Discussion

One goal of public health surveillance for Chagas disease in the United States is to identify local vectorborne transmission and inform strategies to prevent human infection. In Latin America, the risk for infection is high because triatomines infest poorly built housing structures, and peridomestic reservoirs are abundant. The risk for autochthonous transmission in the United States is considered low because of better housing conditions and a lack of transmission associated with domestic reservoirs, such as dogs, and human Chagas cases (*1*,*6*). With a low risk for local transmission and infrequently reported cases of acute infection, there are fewer opportunities for public health response (*1*).

With an estimated 63–315 congenital *T. cruzi* infections occurring annually in the United States (*5*), focused surveillance efforts might be beneficial to identify congenital cases. Timely recognition of infection and treatment will prevent disease development in infected infants and reduce the risk for further transmission (*7*). However, surveillance for congenital Chagas disease is challenging in the absence of routine prenatal or newborn screening. More research is needed to better define groups at risk for transmitting congenitally and to understand how to implement effective screening programs (*1*). These states investigate reported cases for possible congenital transmission, but there are no separate surveillance efforts focused solely on congenital transmission.

Awareness of Chagas disease as a public health problem in the United States increased after the introduction of blood donor screening for Chagas disease in 2007 (*8*). As of December 2017, at least 2,300 infected blood donors had been reported by blood banks across the United States (*9*). Blood donor screening facilitates recognition and treatment of chronically infected patients and serves as an important source of reported cases for surveillance. However, the rate (of positivity) derived from screening of donors underestimates the underlying prevalence of infection in the United States because of the relatively low rates of blood donation among foreign-born Latinos, who are more likely to be infected than are non-Hispanic whites and African Americans (*10*).

The findings in this report are subject to at least one limitation. The data used for this report might have been subject to recall bias because of the time between surveillance implementation activities in each state and study interview.

If resources are available, surveillance for Chagas disease might be important to conduct in states with large populations at risk, including frequent travelers from countries where the disease is endemic and states at risk for local autochthonous transmission (e.g. have infected mammalian reservoirs and appropriate triatomine vectors), to delineate the actual prevalence of disease. Surveillance efforts can also help to increase awareness among providers, identify unmet health care needs for patients, and assist in linking patients with Chagas disease to treatment to help prevent cardiac and gastrointestinal complications. In addition, although the risk for transmission from mother to child is low in the United States, monitoring for congenital Chagas disease might be considered in states with communities at risk.

**Summary**

**What is already known about this topic?**

Most of the estimated 300,000 cases of Chagas disease (caused by *Trypanosoma cruzi* infection) in persons living in the United States were acquired in countries where the disease is endemic.

**What is added by this report?**

In 2017, Chagas disease was reportable in six states. Most cases identified, including among blood donors, are chronic cases and are not the result of local vectorborne transmission.

**What are the implication for public health practice?**

Chagas disease surveillance remains important in states with frequent travelers from countries where the disease is endemic and with a risk for local transmission. Surveillance activities help increase awareness among public health professionals and physicians and can help link persons with chronic Chagas disease to treatment.

## Footnotes

* https://www.cdc.gov/parasites/chagas/gen_info/detailed.html.
